# Fully printed and multifunctional graphene-based wearable e-textiles for personalized healthcare applications

**DOI:** 10.1016/j.isci.2022.103945

**Published:** 2022-02-18

**Authors:** Md Rashedul Islam, Shaila Afroj, Christopher Beach, Mohammad Hamidul Islam, Carinna Parraman, Amr Abdelkader, Alexander J. Casson, Kostya S. Novoselov, Nazmul Karim

**Affiliations:** 1 Centre for Print Research (CFPR), University of the West of England, Frenchay, Bristol BS16 1QY, UK; 2 Department of EEE, University of Manchester, Oxford Road, Manchester M13 9PL, UK; 3 Department of Design and Engineering, Bournemouth University, Dorset, BH12 5BB UK; 4 Department of Materials Science and Engineering, National University of Singapore, Singapore, Singapore; 5 Institute for Functional Intelligent Materials, National University of Singapore, Singapore 117575, Singapore; 6 Chongqing 2D Materials Institute, Liangjiang New Area, Chongqing 400714 China

**Keywords:** Health technology, Materials science, Nanomaterials

## Abstract

Wearable e-textiles have gained huge tractions due to their potential for non-invasive health monitoring. However, manufacturing of multifunctional wearable e-textiles remains challenging, due to poor performance, comfortability, scalability, and cost. Here, we report a fully printed, highly conductive, flexible, and machine-washable e-textiles platform that stores energy and monitor physiological conditions including bio-signals. The approach includes highly scalable printing of graphene-based inks on a rough and flexible textile substrate, followed by a fine encapsulation to produce highly conductive machine-washable e-textiles platform. The produced e-textiles are extremely flexible, conformal, and can detect activities of various body parts. The printed in-plane supercapacitor provides an aerial capacitance of ∼3.2 mFcm^−2^ (stability ∼10,000 cycles). We demonstrate such e-textiles to record brain activity (an electroencephalogram, EEG) and find comparable to conventional rigid electrodes. This could potentially lead to a multifunctional garment of graphene-based e-textiles that can act as flexible and wearable sensors powered by the energy stored in graphene-based textile supercapacitors.

## Highlights

• Graphene-based screen-printed conductive, flexible, and machine-washable e-textiles
• Able to capture movements demonstrating their potential as activity sensors
• In-plane all-solid-state printed textiles supercapacitor showing comparable performance
• Perform as EEG bio-signal electrode comparable to current rigid clinical electrodes

## Graphical abstract

**Figure:**
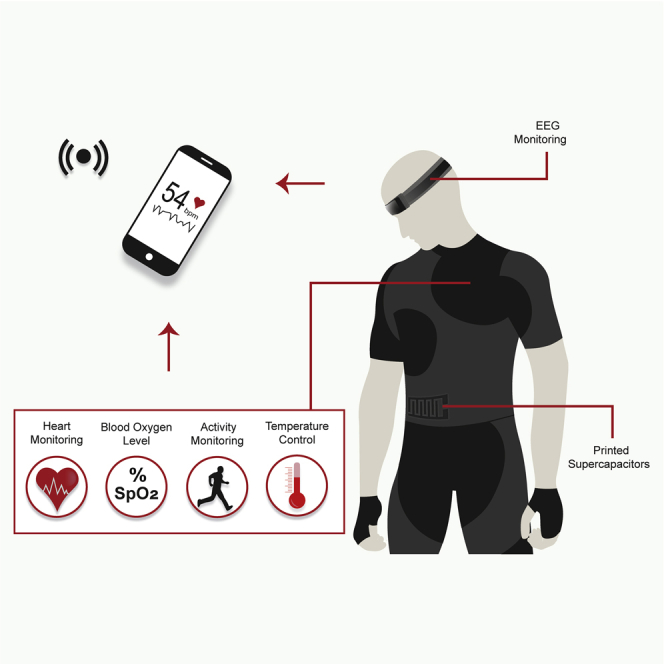

## Introduction

Electronic textiles (e-textiles) have emerged as a new class of flexible wearable devices that could interface with the human body, and continuously monitor, collect, and communicate various physiological parameters (Cao et al., 2018; Iqbal et al., 2021; Lin et al., 2020). Unlike existing bulky and rigid wearable devices, e-textiles are lightweight, comfortable and durable, as well as maintain the desirable electrical properties (Komolafe et al., 2019; Vu et al., 2021; Yao et al., 2019). Multifunctional wearables that can detect and distinguish multi-stimuli, and collect a diverse array of signals using a single device, are of great interest for monitoring physiological conditions (Hozumi et al., 2021; Li et al., 2021; Lian et al., 2020). However, the realization of such wearables remains challenging because existing wearable interfaces are limited in terms of performance, scalability, comfortability, and cost (Afroj et al., 2021b; Kim et al., 2019; Wu et al., 2016).

E-textiles that can capture bio-signals, coupled with flexible energy storage devices, constitute a key breakthrough for personalized healthcare applications (Karim et al., 2020; Meng et al., 2020; Zhang et al., 2019). The monitoring of human activities including brain activity (electroencephalogram, EEG), heart (electrocardiogram, ECG), muscle (electromyogram, EMG), and eye movement (electro-oculography, EOG) has widely been used for medical diagnosis (Fayyaz Shahandashti et al., 2019; Sawangjai et al., 2020; Yuce et al., 2008). Such activities can directly be measured using dry or wet electrodes from low-level ion current (i.e. bio-signals) that is already present in human body parts (Fayyaz Shahandashti et al., 2019). Most commonly used electrodes are wet silver chloride (Ag/AgCl) (Chlaihawi et al., 2018). However, such electrodes require time-consuming skin preparation, as well as a gel to maintain low interface impedance, which limits their performance for long-term biopotentials monitoring, due to leaking out and dehydration of the gel, sweat causing short circuits between two adjacent electrodes, and degraded signal quality over an extended time (Wang et al., 2017). Additionally, the gel can create skin irritation and damage, and may even trigger allergic reactions (Peng et al., 2016; Wu et al., 2021). Textiles-based dry electrodes offer attractive alternative (Beach et al., 2018), but are yet to achieve acceptance for medical use (Meziane et al., 2013).

The widespread applications for wearable e-textiles have been limited by the lack of lightweight, flexible, and high-performance power supply units (Sundriyal and Bhattacharya, 2020). The existing rigid and bulky energy storage devices hardly resemble a regular fabric in terms of hand feel, thickness, or drape (Jost et al., 2013). To realize next generation multifunctional wearable e-textiles, there remains a need for highly functional but small, flexible and comfortable energy storage devices (Kim et al., 2017; Ren et al., 2021). Flexible textiles supercapacitors (SCs) are promising due to their excellent lifetime, lightweight nature, high power density, and ability to deliver even under mechanically deformed conditions (Huang et al., 2016). However, the challenge to achieving wearable textiles supercapacitors lies within insufficient energy density, complex and time-consuming manufacturing process, and poor performance with electrode materials (Sun et al., 2020).

Graphene-based e-textiles have received significant interests in recent years for wearable electronics applications, due to graphene’s extremely high specific surface area, high thermal and electrical conductivity, and excellent mechanical properties (Bhattacharjee et al., 2019, 2020; Islam et al., 2021; Karim et al., 2021b; Wang et al., 2020). Graphene-based textiles could potentially provide a multifunctional platform for manufacturing next generation highly innovative and intelligent e-textile garments that can perform as energy generators and storage devices, sensors, actuators, and electrodes for bio-signal detection, all at the same time (Afroj et al., 2019, 2021a, 2021b; Karim et al., 2021a; Miao et al., 2019). While several scalable techniques were reported to produce graphene-based e-textiles, screen printing in particular is a versatile, highly scalable, and cost-effective technique that has already been exploited commercially for the fabrication of biosensors and chemical sensors (Zhang et al., 2016). Previous studies reported screen printing of graphene-based active materials on textile substrates to fabricate wearable electronics (Abdelkader et al., 2017; Qu et al., 2019; Xu et al., 2019); however, such devices are limited in terms of performance and multifunctionality.

Here, we report a strategy that exploits highly conductive, flexible, and machine-washable graphene-based e-textiles capable of capturing bio-signals and storing energy to develop next-generation multifunctional wearable garments. A simple and scalable screen printing of graphene-based ink on textiles, and subsequent fine encapsulation of conductive track, enables highly conductive and machine-washable e-textiles. Such wearable e-textiles, when bent and compressed in forward and backward directions, demonstrate high flexibility via repeatable response in the electrical resistance change. Additionally, they were able to capture movements of different body parts such as index finger, wrist joint, elbow, and knee joint for demonstrating their potential as such activity sensors. The ink was used to print in-plane electrodes for all-solid-state supercapacitors that showed excellent performance in flexible and non-flexible devices. Finally, the textiles were used to capture EEG bio-signals, offering comparable performance to standard commercial Ag/AgCl electrodes, demonstrating their ability to provide a more comfortable biosensor to current rigid clinical electrodes.

## Results and discussion

### Fully printed and highly conductive graphene-based e-textiles

**Figure 1:**
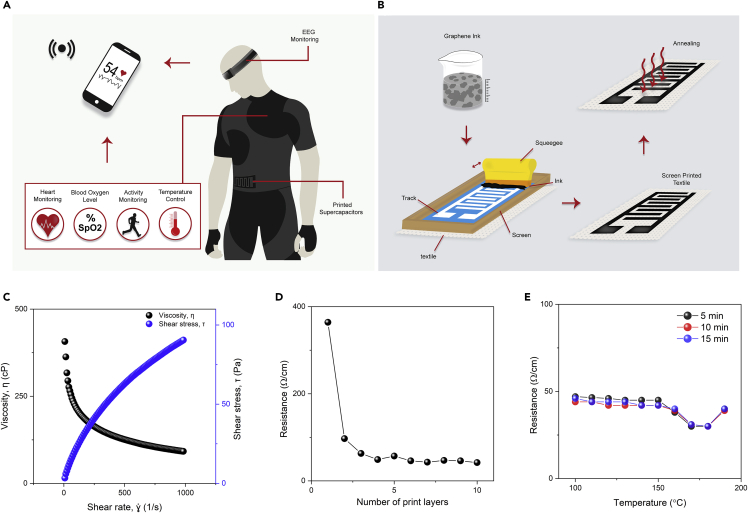
Fully printed and multifunctional wearable e-textiles (A) Schematic diagram of multifunctional aspect of printed e-textiles. (B) Schematic diagram of the screen-printing process for preparing graphene-based wearable e-textiles. (C) Rheological properties of the graphene-based ink, viscosity, and shear stress expressed as a function of shear rate. (D) The change in electrical resistance with the number of print layers for graphene-based ink-printed cotton fabric. (E) The change in electrical resistance of graphene-based ink-printed cotton fabric with curing time and temperature.

Figure 1A illustrates the concept of fully printed and multifunctional wearable e-textiles that monitor vital signs including heart rate, temperature, oxygen saturation level and human activities, as well as store energy via flexible textiles supercapacitors. Such e-textiles can be produced via printing highly conductive graphene-based inks using widely used scalable and high-speed screen-printing technique, Figure 1B. The printed patterns on textiles, when placed on several body parts, act as sensors for collecting and monitoring several physiological information. The collected information from various sensors can then be transferred to a remote data management system wirelessly, providing the opportunity for remote monitoring of physiological parameters of adult patients, children, or people who are elderly. Additionally, the flexible supercapacitor offers an attractive alternative to existing rigid and bulky batteries for powering various wearable devices.

An ideal screen-printing ink is pseudoplastic, which demonstrates shear-thinning properties as its viscosity decreases with the increase of shear rate, allowing the ink to flow from the mesh when shear is applied, and a rapid recovery once shear is removed to yield a high-resolution trace during the separation (Hu et al., 2018). Figure 1C shows the change of viscosity from ∼407.10 cP to ∼92.02 cP over a shear rate up to 1000 s^−1^ for microfluidized graphene ink used in this study. The shear stress also increases up to ∼90.49 Pa over the same shear rate, exhibiting the suitability of the graphene-based ink for screen printing. Most favourable technological effects are obtained when the screen-printing inks possess shear thinning with slight thixotropy (Izak et al., 2017). In agreement to this, the small, enclosed area of the hysteresis loop (Figure S1) of the print ink also supports the suitability of the graphene-based ink for screen printing.

A simple but highly scalable and most widely used screen-printing technique was used to print graphene-based conductive pattern on textiles to produce highly electrically conductive e-textiles. The number of print layers was optimized. After the first print layer, the electrical resistance of the printed fabric was found to be ≈ 364 Ω cm^−1^, Figure 1D. The electrical resistance reduces significantly to ≈97 Ω cm^−1^ after the second print layer and continues to decrease steadily up to four print layers to ≈49 Ω cm^−1^ due to the permeation of an increased amount of graphene ink on the textile surface. After four print layers, the resistance reaches a saturation point and becomes steady as observed until ten print layers. Therefore, four print layers were used to prepare graphene-based printed e-textiles for subsequent processes. All the samples were dried at 100°C for 10 min after each print layer during the print-layer optimization process. The curing temperature and time were then optimized for graphene-printed fabrics. Figure 1E shows that the electrical resistance of the graphene-printed fabric decreases with the increase of curing temperature. The phenomenon could be attributed to the increase of contact force between conductive fillers present in the conductive ink and the shrinkage of the organic binder present in the ink, resulting in a reduced electrical resistance of the print pattern (Koshi et al., 2020). It is worth noting that after applying ∼180°C curing temperature, the cotton fabric turns into a yellow color, as well as the electrical resistance increases, possibly due to the degradation of fiber structures, intra-macromolecular crosslinking, and depolymerization (Afroj et al., 2020). Therefore, we used 170°C for 5 min as curing conditions for all graphene-based printed samples produced for subsequent processes. We further assessed the effect of curing treatment on fabric properties. The annealed substrate exhibited a change in breaking force of only 2.79% in comparison with the untreated substrate, which is very negligible (Table S1 and Figure S2).

### Machine-washable and ultra-flexible graphene-based e-textiles

**Figure 2:**
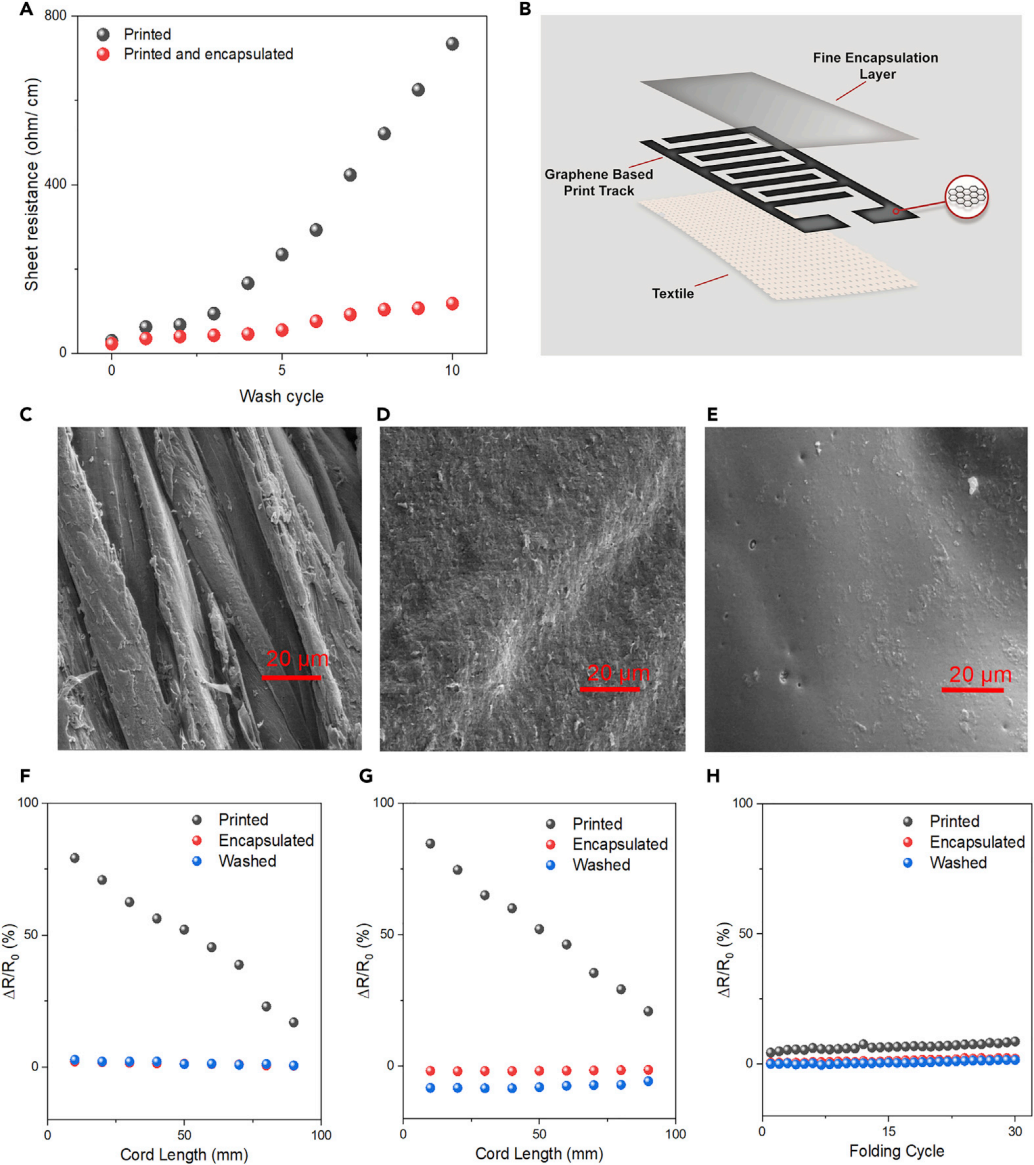
Wash stability and flexibility of printed and encapsulated graphene e-textiles (A) The change in electrical resistance with the number of washing cycles of graphene-based ink printed (without encapsulation) and graphene-based ink-printed (with encapsulation) cotton fabric. (B) Graphical illustration of graphene-based ink pattern and encapsulation layer on textile substrate. (C) Scanning electron microscope (SEM) image of control cotton fiber (X2000). (D) SEM image of graphene-based ink-printed (4 layers) cotton fiber (X2000). (E) SEM image of graphene-based ink-printed (4 layers) cotton fiber (with encapsulation) after washing (10 washing cycles) (X2000). (F) The variation in resistance of the bending sensor in forward direction. (G) The variation in resistance of the compression sensor in forward direction. (H) The variation in resistance under 30 inward (printed pattern inside) folding−releasing cycles.

The poor stability of repeated laundry washings is considered to be one of the major challenges for wide commercial adoption of e-textile products (Rotzler et al., 2020). A good washability is essential for e-textiles to survive intense mechanical deformations and water invasion of washing cycles used during their life cycles (Niu et al., 2021). The washing stability of our graphene-printed e-textiles was assessed following a British Standard (BS EN ISO 105C06 A1S)(ISO, 2010) to evaluate their performance at 10 simulated home-laundry washing cycles. The printed e-textiles patterns (with four print layers) start to lose electrical conductivity just after one washing cycle. The electrical resistance of washed e-textiles was found to be 62.5 Ω cm^−1^ (Figure 2A) after the first washing cycle, which is double than that of the unwashed sample. After repeated washing cycles, the electrical conductivity of such e-textiles is reduced drastically, as the resistance increases significantly to 734.0 Ω cm^−1^ after 10 washing cycles, which is ∼10 times higher than the first washing cycle, Figure 2A. A significant variation in the resistance was also observed at various locations of the washed sample’s surface. The significant increase in the resistance of non-encapsulated samples could be explained by their more proneness to delamination due to the mechanical forces experienced during washing cycles, resulting in losses of electrical conductivity.

The wash stability of wearable e-textiles can be improved via a number of methods including a textile surface pre-treatment with BSA, or by a post-treatment for instance embedding with polydimethylsiloxane (PDMS), polyurethane (PU) sealing, a screen-printed PU top layer, and transferred mold and hot melt encapsulation to seal conductive track on the textile surface (Afroj et al., 2020). Here, a translucent, thin, and stretchable PU-based encapsulant was used to protect graphene-printed wearable e-textiles. Such encapsulation material adheres with textile materials, keeping the printed graphene layer attached to textiles but covered and protected, Figure 2B. The wash stability performance of graphene-printed textiles after encapsulation was then evaluated, where a slight linear increment in electrical resistance was observed after each washing cycle, Figure 2A. The encapsulated graphene-printed e-textiles show ≈1.5 times higher resistance (35.2 Ω cm^−1^) after the first washing cycle in comparison to the unwashed sample. The resistance increases to 118.0 Ω cm^−1^ after 10 washing cycles, which is only about 3.5 times higher resistance in comparison to the first wash cycle.

Figure 2C shows smooth and featureless scanning electron microscope (SEM) of un-treated cotton fibers, which was covered with randomly oriented graphene-based flakes after printing highly concentrated graphene inks, Figure 2D. Additionally, unlike graphene oxide (GO), reduced graphene oxide (rGO) or other functionalized graphene derivatives, graphene flakes in microfluidized graphene inks do not create chemical bonding with cellulosic fibers, as they are mainly dominated carbon in their structure without any oxygen-containing functional groups (Afroj et al., 2020). When subjected to mechanical action during washing cycles, such randomly oriented flakes of printed e-textiles are removed, Figure S6C. Therefore, the electrical resistance of graphene-printed e-textiles decreases significantly after each washing cycle. However, the encapsulated printed fabric surface exhibits more resistance to delamination due to the protection of the graphene-printed pattern with thin PU layer, Figure S6D. Thus, the wash stability of graphene-printed e-textiles improved substantially via fine encapsulation with a PU layer without any negative effect on the hand feel as well as flexibility of the conductive e-textiles.

The flexibility of printed, encapsulated, and washed graphene-printed e-textiles was also evaluated, Figures 2F and 2G. Graphene-printed (4layers) and encapsulated cotton fabrics were tested both before and after 10 washing cycles. The change in their electrical resistances per 10 cm length due to bending, compression (both in forward and backward direction) was measured. The cord length, which was measured by the grip distance of the sample ends during the experiment (Figure S7), changed (10–90 mm) when the fabrics were bent and compressed both in forward and backward directions. Figures 2F and 2G exhibit repeatable responses in the change of resistance (ΔR/R_0_) during bending and compression in forward direction and backward directions, Figures S8 and S9, respectively. However, the encapsulated samples show excellent repeatability in comparison with the unencapsulated samples for both before and after washing operations. Additionally, the variation of resistances was found almost stable for both unwashed and washed samples subjected to 30 inward (Figure 2H) and outward (Figure S10) folding−releasing operations. It is worth noting that no visible changes in appearance or shape or creasing were observed due to those mechanical actions (bending, compression, and folding cycles), demonstrating excellent flexibility and bendability of encapsulated and washable graphene-based wearable e-textiles.

### Activity monitoring wearables of graphene-based e-textiles

**Figure 3:**
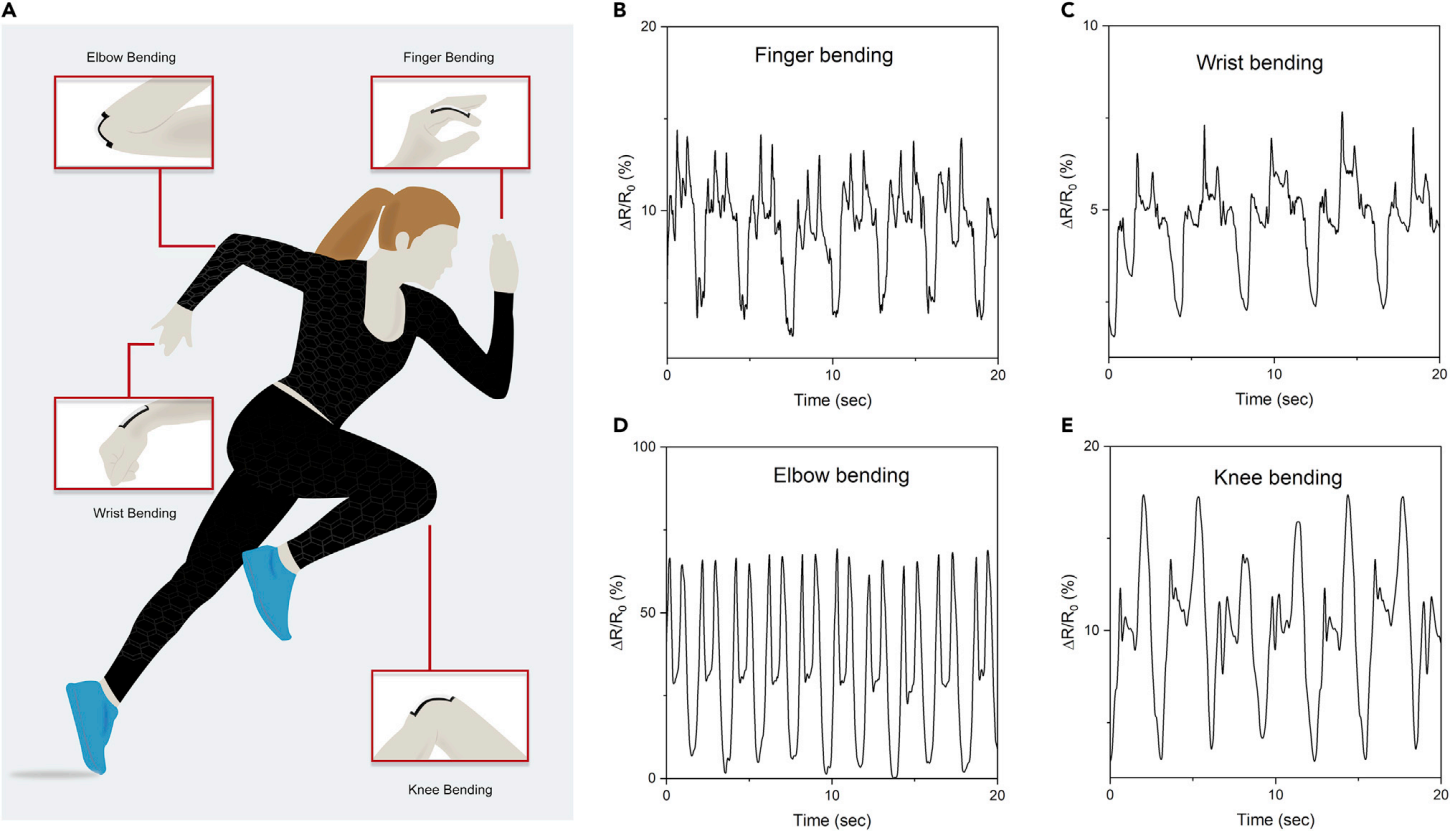
Printed graphene e-textiles as activity monitoring sensors (A) Schematic diagram showing the application of graphene-based ink-printed textiles as activity monitoring sensors at different body parts. The motion detection represented by the change of resistance as a function of time during (B) finger joint bending (C) wrist joint bending (D) elbow joint bending and (E) knee joint bending by the graphene-based ink-printed textiles.

Wearable sensors for monitoring individual’s as well as patient’s health conditions via gathering physiological and movement data have received significant attention now-a-days due to their continuous and non-invasive nature (Karim et al., 2017a; Teymourian et al., 2020; Wang et al., 2019). The strain-type sensors, composed of conductive network of active materials, serve as resistor under applied voltage, the mechanism of which is known as piezoresistivity. During stretch/compression, the electrical resistance of the conductive track changes as function of the applied mechanical strain that originates from the geometrical changes such as length and/or cross-sectional area, intrinsic resistive response of active materials, tunneling effect, and/or disconnection mechanism. The resistance recovers to its initial values in a reversible manner after releasing from the applied strain. The deformation state, thus, can be readily measured by recording the changes in the electrical resistance of the resistive-type strain sensors (Souri et al., 2020). Graphene-printed conductive, washable, and flexible e-textiles were attached on different body parts such as index finger, wrist joint, elbow, and knee joint to demonstrate their potential as activity sensors, Figure 3A. The change of the resistances with the movement of such body parts were measured (Figures 3B–3E). It is noteworthy here; we utilized the similar printed patterns as piezoresistive sensors at different body parts. Although similar patterns were used, the sensors placed at different body parts such as finger, wrist, elbow, and knee joints were exposed to different stress due to physical movements for different body parts. These produced different strains on the sensors, therefore the resulted repeatable responses were different for different body parts. Repeatable responses of the change of resistances were observed in all the cases over time with outstanding capability of capturing various mechanical actions. This is in agreement with previous study where similar repeatable response and excellent capability of sensitivity measurements were achieved for both unwashed and washed (10 times) skin-mounted strain sensors (Afroj et al., 2020). Such sensors could be used to monitor physical activities such as walking, eating, running, brushing etc of patients or people who are elderly on daily basis and send to their family members or carers to inform about their health status to help them to live independent safely (Al-Khafajiy et al., 2019, Kabiri Ameri et al., 2017). This approach is also effective to study patient’s behavioral changes and recovery processes while still living at their homes.

### Printed flexible energy storage e-textiles

**Figure 4:**
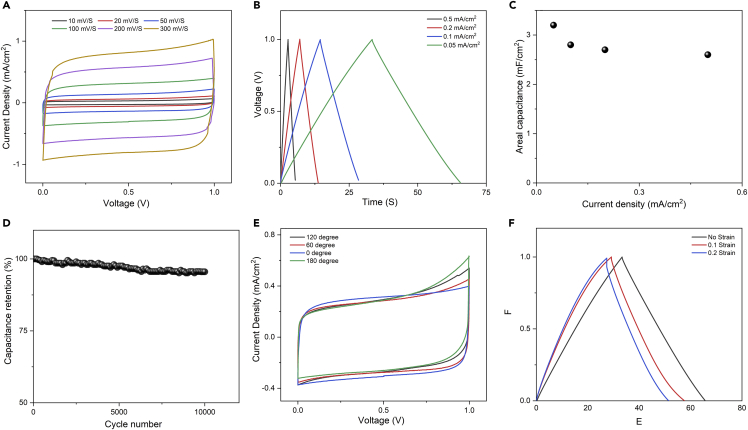
Characterization of graphene-based ink-printed textile supercapacitor (A) CV curves at multiple scan rates, (B) Charge/discharge curves at various current densities, (C) The change of the areal capacitance with the current density, (D) The cyclic stability of the printed supercapacitor measured at 0.1 mA cm^−1^, (E) CV of the printed supercapacitor at different bending angles, (F) Charge/discharge profile for the supercapacitor with no strain and under strain.

We have also printed a solid-state symmetrical supercapacitor device on the textile using the same screen-printing technique. The printed device has five branches on both the cathode and the anode sides; each branch is 2 mm in width and 33 mm in length (Figure S14). The distance between each branch on the cathode and the neighbor branch from the anode is 2 mm. The electrochemical performance of the in-plane supercapacitor was assessed by cyclic voltammetry (CV) and galvanostatic charge-discharge. As can be seen in Figure 4A, the CV curves show almost a rectangular shape at all tested scan rates, indicating ideal double-layer capacitance. The lack of any redox peak in the tested electrochemical windows suggests that graphene-based ink is of very good purity, and completely cover the cotton fibers. To calculate the areal capacitance, the charge-discharge profile was recorded at current densities ranging from 0.05 to 0.5 mA cm^−2^ (Figure 4B). In agreement with the CV results, the charge-discharge profiles exhibit an ideal double-layer behavior with a triangular shape and equal time for charge and discharge. Also, there are no visible plateaus or bends in the profiles that might be attributed to a redox reaction. The lack of any IR (I = current and R = inner resistance) drops at the beginning of the discharge curve suggests good conductivity and low charge barriers at the electrodes. The maximum calculated areal capacitance of the symmetrical printed supercapacitor was 3.2 mF cm^−2^, which is comparable with many reports existing in literature (Table 1). Even when the current density increased by 10 times to 0.5 mA cm^−2^, an aerial capacitance of 2.6 could be maintained, equivalent to capacitance retention of 81.3% (Figure 4C). The electrochemical stability of the supercapacitor was investigated based on long-term charge-discharge curves at a current density of 0.1 mA cm^−2^. The devices lost only 5% of their initial capacitance after 10,000 cycles, indicating excellent stability (Figure 4D). Without using any current collector or conductive agents, the printed supercapacitor could deliver a high areal energy density of 0.28 mWh cm^−2^ at the power density of 3 mW cm^−2^.

**Table 1:** Comparison of the electrochemical performance of the graphene-ink printed energy storage textile with others reported in the literature

Assembly of energy storage textiles	performance	Energy and power density	Device retention	Flexibility	Application	Ref
Screen-printed rGO on cotton followed by reduction with PVA/H_2_SO_4_ solid electrolyte	Areal capacitance 2.5 mFcm^−2^	–	97% after 10,000 cycle	95.6% after folding 180° for 2000 cycles	Supercapacitor	(Abdelkader et al., 2017)
Graphene film with PVA/H_2_SO_4_ solid electrolyte	2.7 mF cm^−2^	–	–	–	Supercapacitor	(Chen et al., 2014)
Stretchable textiles fully printed Ag@PPy@MnO_2_ on Ag cathode electrode and activated carbon on Ag anode electrode with PVA/Na_2_SO_4_ electrolyte	426.3 mF cm^−2^ (cathode)	0.0337 mWh cm^−2^ at 0.38 mWcm^−2^	90.8% retention after 5000 cycles	86.2% retention after 40% stretching strain	Supercapacitor	(Liu et al., 2018)
PPy electrochemically deposited on rGO-painted SnCl_2_ modified polyester textiles with PVA/H_2_SO_4_ gel electrolyte	1117 mF cm^−2^ at a current density of 1 mA cm^−2^	0.0658 mWh cm^−2^ at 1 mA cm^−2^ and 0.5 mW cm^−2^	100% after 10,000 cycles	98.3% after 1000 bending cycles	Supercapacitor	(Li et al., 2018)
Coating of poly-cotton textiles with graphene ink	Resistance 11.9 Ωsq^−1^, Areal capacitance 2.7 mF cm^−2^	–	98% after 15,000 cycles	98% after 150 cycles of bending at 180°	Activity monitoring sensor and Supercapacitor	(Afroj et al., 2020)
Kevlar fibers, coated in gold, and then grew ZnO nanowires with PVA/H_3_PO_4_ solid electrolyte	Areal capacitance 2.4 mF cm^−2^	2.7 × 10^−5^ mWhcm^−2^	–	–	Supercapacitor	(Bae et al., 2011)
CNT on Ti wire with PVA/H_2_SO_4_ solid electrolyte	Areal capacitance 1.84 mF cm^−3^	0.16 × 10^−3^ mW h cm^−3^ and 0.01 mW cm^−3^	80% after 1000 cycles	–	Supercapacitor	(Chen and Dai, 2016)
SnS/S-doped graphene on PET with PVA/H_2_SO_4_ solid electrolyte	Areal capacitance 2.98 mF cm^−2^	–	99% after 10,000 cycle	–	Supercapacitor	(Liu et al., 2017)
N-Doped rGO on PET with PVA/H_3_PO_4_ solid electrolyte	Areal capacitance 3.4 mF cm^−2^	0.3 mWh cm^−3^ at 0.2 W cm^−3^	98% after 2000 cycles	–	Supercapacitor	(Liu et al., 2014)
Graphene ink screen printed on cotton textiles with PVA/H_2_SO_4_ gel electrolyte	Resistance 30 Ω cm^−1^, Areal capacitance 3.2 mFcm^−2^	0.28 mWh cm^−2^ at 3 mW cm^−2^	95% after 10,000 cycles	–	Activity monitoring sensor, EEG electrode, Supercapacitor	This work

For the supercapacitor to be a part of an integrated device/system on a smart textile, it is important to evaluate the performance of the printed device under various loading and strains in multiple directions. We first assessed the performance of the printed supercapacitor using a bending test at various angles. The CV curves were recorded at different bending angles (Figure 4E), which show minor changes of the original unbent curve, suggesting good mechanical stability under bending. Figure 4F shows the charge-discharge curves of the supercapacitors with no strain and under the biaxial strain of 0.1 and 0.2 strains at a constant current density of 0.1 mA cm^−2^. Interestingly, the charge-discharge profiles of the supercapacitor under strain exhibit some bending toward the end of the discharge. We expected this bending to occur due to a partial exposure of the substrate cotton fibers to the supercapacitor electrolyte. It is known that the cotton fiber’s surface is rich with oxygen functional groups, which might introduce some faradic reaction. Obviously, the contribution of such redox reactions is limited due to the limited exposure of the fiber. There is a noticeable potential IR drop at the beginning of the discharge curve when subjected the electrodes to strain, which increases by increasing the strain. We believe the increase of the electrode resistivity is related to the loss of the graphene flakes under strain, which demonstrates the possibility of using printed graphene electrodes as strain and piezoelectric sensors.

### Sleep monitoring via electroencephalography recordings (EEG)

**Figure 5:**
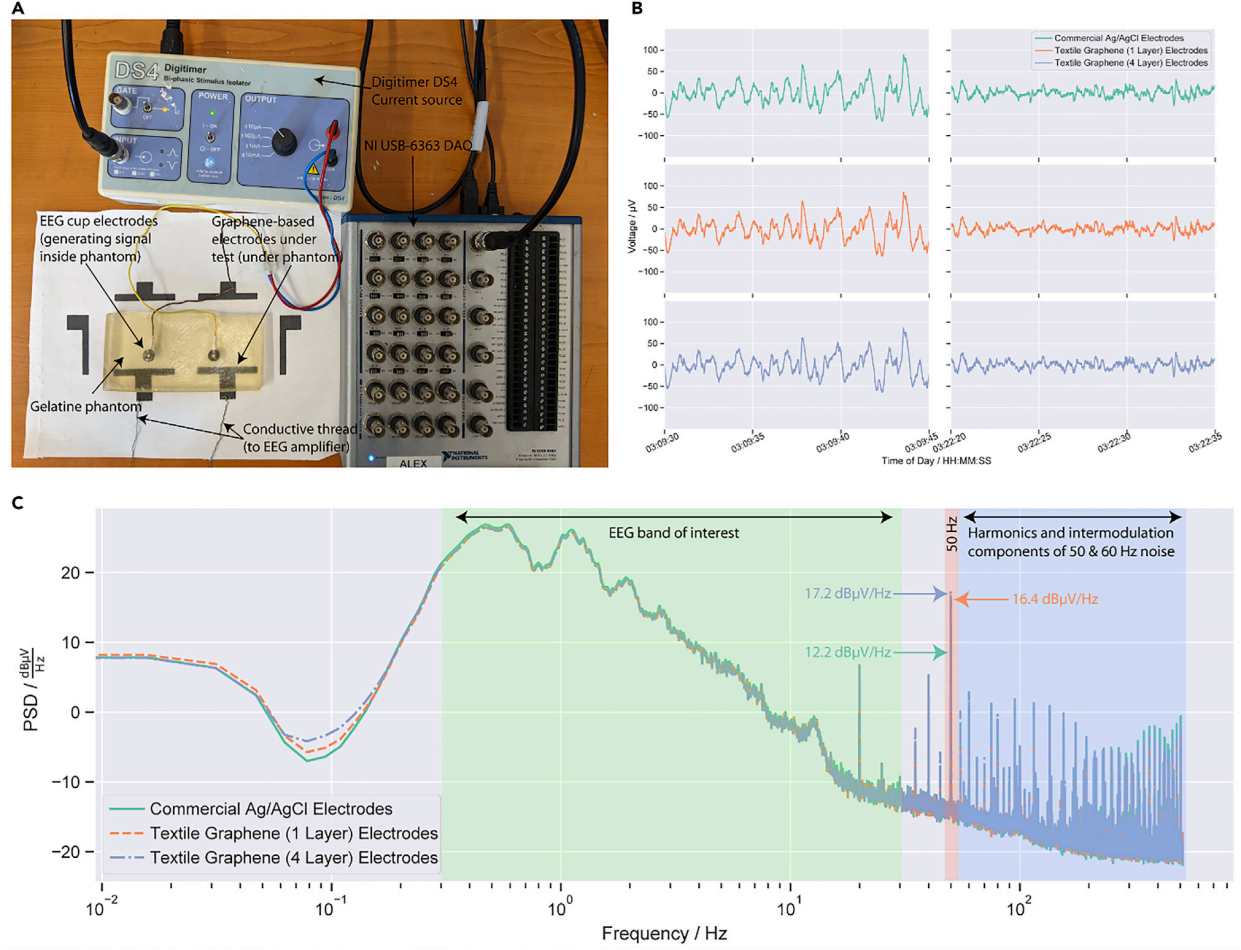
Printed graphene e-textiles for EEG applications (A) Experimental setup showing signal source (DAQ), current stimulator (Digitimer DS4), gelatine phantom, graphene-based electrodes, and conductive thread connection to EEG Amplifier. (B) Data collected using the textile graphene-based electrodes against commercial Ag/AgCl electrodes from data from Fpz-Oz data in the SC4001 record from Physionet. (left) A section of the record in deepest stage of sleep (stage 4), indicated by the presence of slow oscillations in the record. (right) A lighter stage of sleep (stage 2), indicated by smaller amplitude higher frequency oscillations. (C) Power spectral density of recorded signal from graphene textiles electrodes against commercial Ag/AgCl electrodes, highlighting the typical frequency band of interest in EEG studies shaded in green. The 50 Hz component and its contribution for each of the electrode types are shaded in red. Also shown are higher frequency harmonics and intermodulation components from 50 to 60 Hz noise shaded in dark blue.

We also demonstrated how our printed graphene-based textile electrodes can be used to record electrophysiological information, specifically the electroencephalogram (EEG). An EEG is a non-invasive method of recording brain activity, undertaken by placing electrodes on the scalp and forehead of a person, and used in a number of areas from sleep studies to epilepsy diagnosis (Biasiucci et al., 2019). Here, we demonstrate how these graphene-based e-textiles can be used as electrodes in sleep monitoring, Figure 5A. Sleep studies which take polysomnographic recordings (PSG), consisting principally of EEG recordings and other bio-signals are recordings taken overnight during sleep, Figure 5B. The PSG data is scored into different sleep stages by a trained expert to identify sleep disorders (Sharma et al., 2021; Yildirim et al., 2019). To demonstrate the feasibility of using the e-textiles for EEG recordings, we simulate the EEG part of a PSG using two electrodes and a gelatine head phantom (Owda and Casson, 2021), Figure 5A. This setup simulates one EEG electrode placed on the central forehead (referred to to as Fpz in the international 10-20 standard), and a reference electrode placed elsewhere (here, we utilize the Cz location, which is in the center of the scalp). This reference electrode could also be positioned in an alternative hairless location, such as behind the ear, but here we use the Cz location to allow standardization with other datasets.

Prior to any pre-processing or filtering of data, we examine the frequency domain of the EEG recordings by comparing the power spectral density (PSD) of the signals collected from the electrodes. This allows identification of the level of 50 Hz noise picked up by the electrodes, a common source of noise in electrophysiological recordings. This 50-Hz pickup is caused by the electrical mains (which oscillates at 50 Hz in the UK) coupling with the electrodes and measurement equipment. In Figure 5C, the PSD is shown, and we can see that over the frequency band of interest for sleep studies (0.3–30 Hz) (Baker et al., 2012) all electrode types follow each other very closely. At 50 Hz, the commercial Ag/AgCl electrodes have around 12.2 dBμV/Hz of power contribution, and the graphene-based electrodes have a slightly higher contribution, around 16.4 dBμV/Hz and 17.2 dBμV/Hz for the 1-layer and 4-layer electrodes, respectively. We can also see higher frequency noise above 50 Hz in all electrodes, consisting of harmonics and intermodulation components of 50 and 60 Hz noise. These components arise from the 50 Hz noise that is coupled to the electrodes during our experiment and the 60 Hz noise that is present in the original EEG dataset used, (60 Hz is the mains line frequency in the US where the original dataset was collected). In this work, not only are the differences in noise between the graphene-based textiles electrodes and rigid Ag/AgCl electrodes minimal but also this noise does not overlap with the EEG frequency bands of interest, so these power line contributions can be filtered out.

The correlation coefficient between the filtered data collected from both types of graphene-based electrodes over the 20-min record is high at over 0.998, indicating a very strong similarity between the performance of our electrodes and the rigid Ag/AgCl electrodes. Figure 5B shows two 15 s segments of the EEG record data, at two distinct points. In the left figure, data are displayed from the participant when they are in the deepest stage of sleep (stage 4), indicated by the large-amplitude slow-wave oscillations. The right figure shows the data from the participant around 10 min later where they are in a lighter stage of sleep (stage 2), indicated by the higher frequency and smaller amplitude oscillations. Both features can clearly be seen both in the data collected by the graphene-based electrodes and by the rigid Ag/AgCl electrodes indicating similar levels of performance between all electrode types. The results indicate these e-textiles could be utilized for EEG recordings which overcome the limitations of conventional rigid Ag/AgCl electrodes.

### Conclusion

A fully printed, highly conductive, flexible, and machine-washable e-textiles platform that can store energy and monitor physiological conditions including bio-signals is reported. The printed conductive textiles show outstanding wash stability even after 10 home laundry washing cycles. The repeatable responses after bending, compression, and folding cycles exhibit the outstanding flexibility of printed e-textiles. Additionally, we demonstrate potential skin-mounted wearable sensors and sleep monitoring applications of such printed e-textiles. Furthermore, an in-plane flexible solid-state supercapacitor device was also fabricated from the printed e-textiles, which was able to provide an aerial capacitance of ∼3.2 mF cm^−2^ and outstanding cyclic stability upto 10,000 cycles. We believe these findings could potentially lead to a truly multifunctional wearable garment for personalized healthcare applications.

## STAR★Methods

### Key resource table

**Table:** 

REAGENT or RESOURCE	SOURCE	IDENTIFIER
**Chemicals, peptides, and recombinant proteins**		

Screen printing ink	Cambridge Graphene	Graphink 2/CMC
Stretchable encapsulant	DuPont	PE773
PVA	Sigma-Aldrich	341584
H_2_SO_4_	Sigma-Aldrich	258105

### Resource availability

#### Lead contact

Further information and requests for resources and reagents should be directed to and will be fulfilled by the lead contact, Nazmul Karim (nazmul.karim@uwe.ac.uk)

#### Materials availability

This study did not generate new unique reagents.

### Method details

#### Materials

A water-based graphene dispersion (100 g/L) was prepared using Microfluidized technique (Afroj et al., 2019; Karim et al., 2018). The rheology of graphene-based dispersions was modified using carboxymethylcellulose sodium salt to prepare printable inks, which was supplied by Versarien Limited UK. Microcircuit encapsulant PE773 was purchased from Dupont (USA). 100% cotton fabric (de-sized, scoured, and bleached which are ready-to-dye fabric) was manufactured at Square Fashions Limited (Bangladesh), and donated by 2dtronics Limited (UK).

#### Screen printing of graphene ink on textiles and encapsulation

A basic hand screen-printing method was used to print graphene-based ink on textiles, and then printed fabric was dried at 100°C for 10 minutes using a small table-top thermo fixation oven (Mini-Thermo, Roaches, UK). Each print cycle consisted of one printing layer (3 passes) followed by drying process. Samples were repeatedly printed and dried to optimize the number of print layers (1–10 layers). Additionally, the curing temperature and time were also optimized using a range of curing temperature (100 °C-190°C with 10°C interval) over a time range (5–15 minutes with 5 minutes interval). For wash stability tests, graphene printed textiles were encapsulated with a fine layer of a microcircuit encapsulant PE773 using the same screen-printing method followed by a drying and curing at 150°C for 1 min.

#### Characterization of graphene ink and wearable e-textiles

The ink viscosity was characterized using a HAAKE Viscotester iQ Rheometer (ThermoFisher Scientific, UK). Shear stress and viscosity were determined at different shear rates to assess the printing ink’s flow properties. The surface topography of the control cotton fabric, graphene-printed fabric, graphene-printed fabric after washing, graphene printed-encapsulated fabric and graphene-printed and encapsulated fabric after washing were analysed using a FEI Quanta 650 field emission scanning electron microscope (SEM).

The electrical resistance of graphene printed textile was measured by a standard multi-meter. The wash stability of graphene printed fabric was carried out according to BS EN ISO 105 C06 A1S method as previously reported (Afroj et al., 2020). During wash stability tests, a conductive track was created with electrically conductive silver paste for measuring the electrical resistance of graphene printed and encapsulated e-textiles, Figure S6B. By following previously reported methods (Afroj et al., 2019; Karim et al., 2017b) various cord lengths were used to measure the change of resistance of washed and unwashed fabric (10 cm × 1 cm strip) during bending (concave down) and compression (concave upward), Figure S7. A Win Test tensile tester (Testometric, UK) was used to control the cord length both in forward and reverse directions for both bending and compression tests. Printed samples were also subjected to ten repeated folding–release cycles in both inward and outward direction. The change in the resistance of skin mounted graphene-printed fabric sensors on index fingers, wrist, elbow, and knee joint was captured using a Keithley digital multi-meter.

#### Supercapacitor device fabrication and electrochemical characterization

The supercapacitor device was prepared by following previously reported method (Abdelkader et al., 2017). The printed graphene textile electrodes were used as the current collector. However, copper sheets were glued to the end of every electrode to ensure good electrical contact with the measuring workstation. The printed electrodes were coated with a hydrogel-polymer electrolyte, poly (vinyl alcohol) (PVA) doped with H_2_SO_4_. The H_2_SO_4_ PVA gel electrolyte was prepared as follows: 1 g of H_2_SO_4_ was added into 10mL of deionized water, and then 1 g of PVA (molecular weight: 89 000–98 000, Sigma-Aldrich) was added. The whole mixture was then heated to 85°C under stirring until the solution became clear. The electrolyte was drop-casted and left to dry overnight under ambient conditions to ensure that the electrolyte completely wetted the electrode and to allow for evaporation of any excess water.

The electrochemical performances of the printed devices were investigated by cyclic voltammetry (CV), and galvanostatic charge/discharge tests. The electrochemical measurements were performed on an Iviumstat Electrochemical Interface. The CV and galvanostatic charge–discharge measures were conducted in the potential range of −0.2 to 0.8 V at different scan rates and current densities. For measuring the CV at different bending angles, the device was attached to a flexible polyethylene terephthalate film.

#### Graphene-based wearable EEG

To verify the ability of our material to capture EEG information, a phantom head model was created using gelatine in a 1:4 ratio by mass of gelatine to water, creating a replication of the ionic conductors present in the human body, as previously reported (Velcescu et al., 2019). The phantom consists of two rigid sintered silver/silver chloride (Ag/AgCl) EEG electrodes embedded inside, enabling pre-recorded EEG signals to be played-back; replicating signals present inside the human head and giving a ‘known’ signal to record, allowing verification. We used pre-recorded EEG data from the Physionet Sleep-EDF Database (Goldberger et al., 2000; Kemp et al., 2000), a dataset consisting of expert-scored PSG sleep study recordings, to generate signals in the phantom. A 20-minute excerpt (03:03:00 – 03:23:00) from participant SC4001 using the Fpz-Oz channel was played back in the phantom using a current source (DS4 Biphasic Stimulus Isolator, Digitimer), set to ±10 μA, in turn connected to a digital-to-analogue voltage source (NI USB-6363, National Instruments, USA). Using silver-loaded conductive thread (Electro Fashion, Kitronik, UK) connections to two of the conductive areas on the textiles were sewn in and in turn connected to an electrophysiological amplifier (Actiwave EEG/ECG 4 channel, CamNtech), sampling at 1024 Hz and 10 bits. The textile electrodes were compared against commercially available gold-standard rigid EEG electrodes (Ag/AgCl disc electrodes and Abralyt HiCl conductive gel) commonly used in EEG studies. Frequency-domain comparisons between electrode types were made by calculating the power spectral density (PSD) using the welch method with 30 s long windows, 50% overlaps and 2^16^ FFT points. Time-domain comparisons were made after filtering with an eight-order zero-phase notch filter with f_L_ and f_H_ set to 47.5 Hz and 52.5 Hz respectively, and then filtered with a fourth-order zero-phase low-pass filter with f_L_ = 50 Hz. The 2D correlation coefficient between the filtered data collected from both types of graphene electrodes was calculated against the commercial rigid electrodes.

## Data Availability

The published article includes all data generated or analyzed during this study.
