# Supplementary material for: Fully printed and multifunctional graphene-based wearable e-textiles for personalized healthcare applications
Source: iScience. 2022 Feb 18;25(3):103945. doi: 10.1016/j.isci.2022.103945 (PMC8914337; doi:10.1016/j.isci.2022.103945)
Supplement: Document S1. Figures S1–S14, Tables S1 and S2 [file mmc1.pdf]

iScience, Volume *25*

Supplemental information

Fully printed and multifunctional graphene-based wearable e-textiles for personalized healthcare applications

Md Rashedul Islam, Shaila Afroj, Christopher Beach, Mohammad Hamidul Islam, Carinna Parraman, Amr Abdelkader, Alexander J. Casson, Kostya S. Novoselov, and Nazmul Karim

Supplemental Figures and Tables

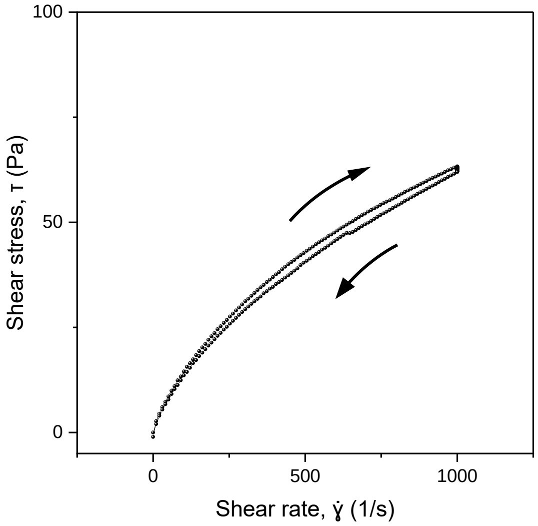

Figure S1. Thixotropic behaviour of the graphene-based print ink, Related to Figure 1.

Table S1. Comparison of tensile properties of annealed substrate in comparison with the untreated substrate according to EN ISO 13934-1, Related to Figure 1.

Parameters	Un-treated substrate	Annealed (at 170°C)	% Change
Breaking force (N)	613.30	596.18	-2.79
Standard deviation	46.73	52.76	-
Elongation (mm)	34.63	33.43	-3.47
Standard deviation	1.55	1.61	-

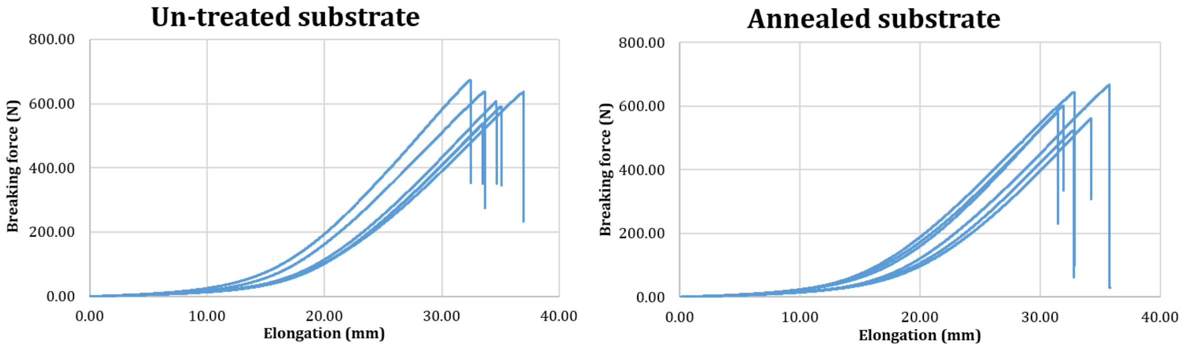

Figure S2. Force-elongation curves for untreated and annealed substrate, Related to Figure 1.

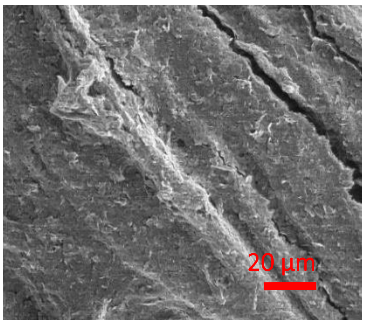

Figure S3

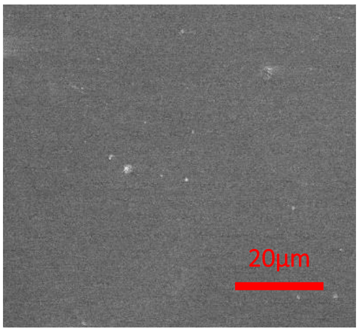

Figure S4

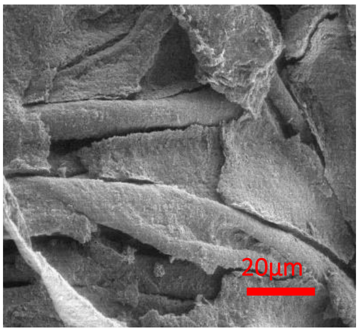

Figure S5

Figure. Scanning electron microscope (SEM) images of **S3.** printed (1 layer) fabric (×2000), **S4.** Printed (4 layer) and encapsulated fabric (×2000), and **S5.** washed without encapsulation of graphene-ink printed cotton fabric (×1000), Related to Figure 2.

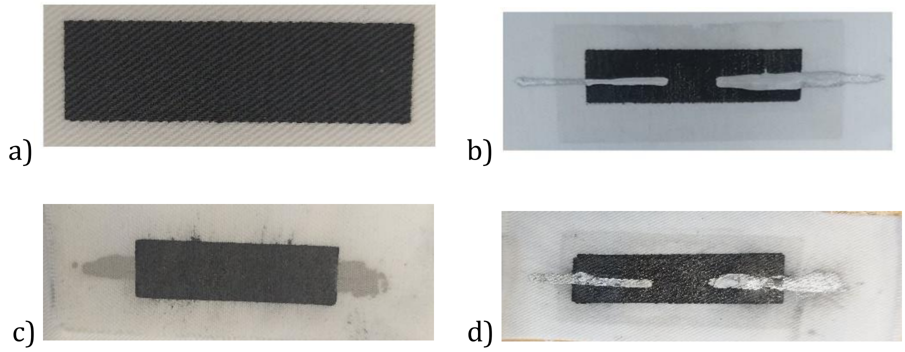

Figure S6. Digital photograph of a) printed (4 layer), b) encapsulated, c) washed without encapsulation, and d) washed after encapsulation of graphene-ink printed cotton fabric, Related to Figure 2.

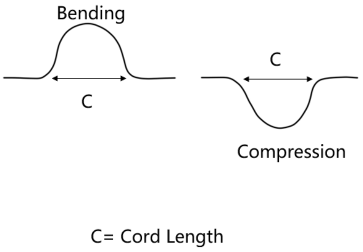

Figure S7. The cord lengths during bending (concave down) and compression (concave upward) of graphene-ink printed textiles**,** Related to Figure 2.

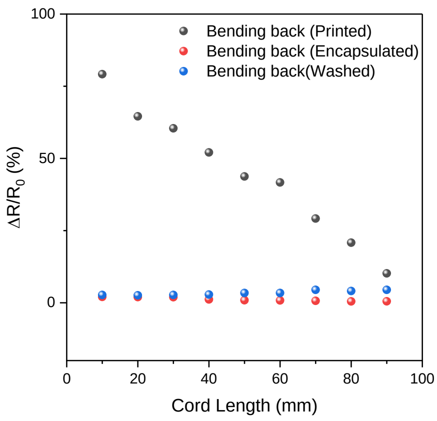

Figure S8. The variation in resistance of the bending sensor in backward (bending back) direction, Related to Figure 2.

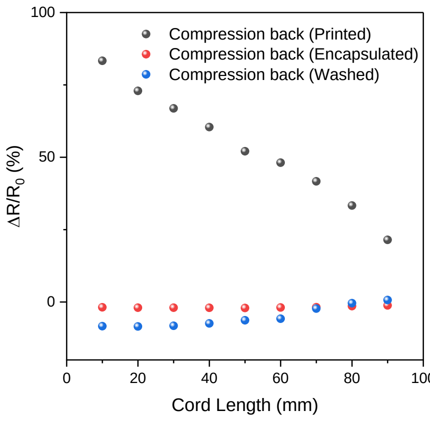

Figure S9. The variation in resistance of the compression sensor in backward (compression back) direction, Related to Figure 2.

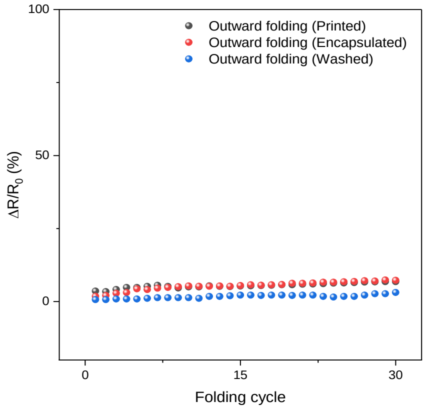

Figure S10. The variation in resistance under 30 outward (printed pattern outside) folding–releasing cycles, Related to Figure 2.

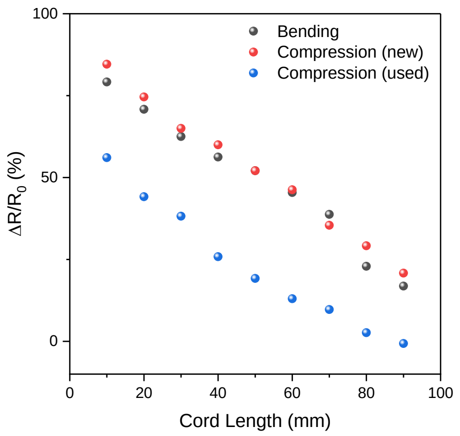

Figure S11. The variation in resistance of the compression sensor (forward direction) while used as new sample versus a bended sample, Related to Figure 2.

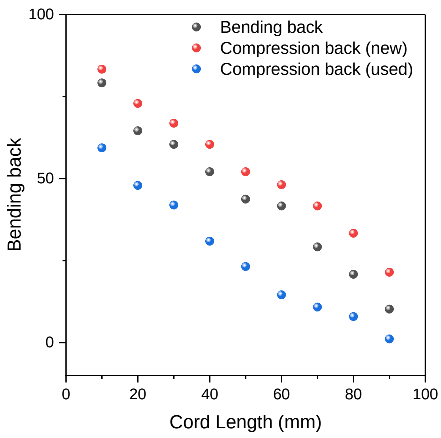

Figure S12. The variation in resistance of the compression sensor (backward direction) while used as new sample versus a bended sample, Related to Figure 2.

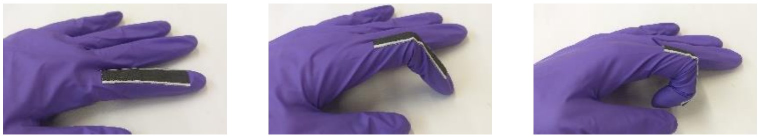

a)

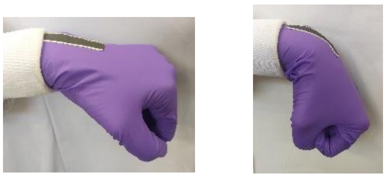

b)

c)

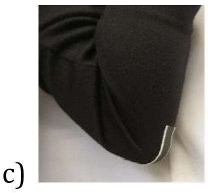

d)

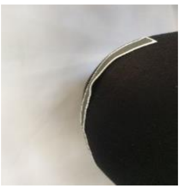

Figure S13. Activity monitoring with the printed sensors a) finger bending b) wrist bending c) elbow bending and d) knee bending, Related to Figure 3.

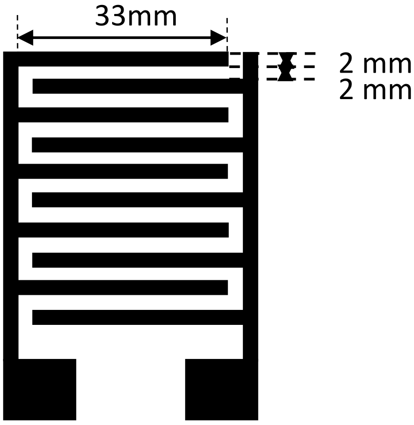

Figure S14. Printed supercapacitor device Related to Figure 4.

Table S2. Comparison of the electrochemical performance of the printed e-textile with others in the literature, Related to Figure 4.

Preparation of e-textiles	performance	Energy and power density	Device retention	Flexibility	Application	Ref.
Screen printed rGO on cotton followed by reduction with PVA/H_2SO_4 solid electrolyte	Areal capacitance 2.5 mFcm^{-2}	-	97 % after 10000 cycle	95.6% After bending	Supercapacitor	(Abdelkader et al., 2017)
Graphene film with PVA/H_2SO_4 solid electrolyte	2.7 mF cm^{-2}	-	-	-	Supercapacitor	(Chen et al., 2014)
Stretchable textiles fully printed Ag@PPy@MnO_2 on Ag cathode electrode and activated carbon on Ag anode electrode with PVA/Na_2SO_4 electrolyte	426.3 mF cm^{-2} (cathode)	0.0337 mWh cm^{-2} at 0.38 mWcm^{-2}	90.8% retention after 5000 cycles	86.2% retention after 40% stretching strain	Supercapacitor	(Liu et al., 2018)
PPy electrochemically deposited on rGO painted $SnCl_2$ modified polyester textiles with PVA/H_2SO_4 gel electrolyte	1117 mF cm^{-2} at a current density of 1 mA cm^{-2}	0.0658 mWh cm^{-2} at 1 mA cm^{-2} and 0.5 mW cm^{-2}	100% after 10 000 cycles	98.3% after 1000 bending cycles	Supercapacitor	(Li et al., 2018)
Coating of poly-cotton textiles with graphene ink	Resistance 11.9 Ωsq^{-1}, Areal capacitance 2.7 mF cm^{-2}		98% after 15 000 cycles	98% after 150 cycles of bending at 180°	Activity monitoring sensor and Supercapacitor	(Afroj et al., 2020)
Kevlar fibres, coated in gold, and then grew ZnO nanowires with PVA/ H_3PO_4 solid electrolyte	Areal capacitance 2.4 mF cm^{-2}	2.7×10^{-5} mWhcm^{-2}		-	Supercapacitor	(Bae et al., 2011)
CNT on Ti wire with PVA/H_2SO_4 solid electrolyte	Areal capacitance 1.84 mF cm^{-3}	0.16×10^{-3} mW h cm^{-3} and 0.01 mW cm^{-3}	80 % after 1000 cycles	-	Supercapacitor	(Chen and Dai, 2016)
SnS/S doped graphene on PET with PVA/H_2SO_4 solid electrolyte	Areal capacitance 2.98 mF cm^{-2}		99% after 10000 cycle	-	Supercapacitor	(Liu et al., 2017)
N-Doped rGO on PET with PVA/ H_3PO_4 solid electrolyte	Areal capacitance 3.4 mF cm^{-2}	0.3 mWh cm^{-3} at 0.2 W cm–3	98% after 2000 cycles	-	Supercapacitor	(Liu et al., 2014)
Graphene ink screen printed on cotton textiles with with PVA/H_2SO_4 gel electrolyte	Resistance 30 Ω cm^{-1}, Areal capacitance 3.2 mFcm^{-2}	0.28 mWh cm^{-2} at 3 mW cm^{-2}.	95% after 10000 cycles	-	Activity monitoring sensor, EEG electrode, Supercapacitor	This work

Supplemental References

Abdelkader, A. M., Karim, N., Vallés, C., Afroj, S., Novoselov, K. S. & Yeates, S. G. 2017. Ultraflexible and robust graphene supercapacitors printed on textiles for wearable electronics applications. *2D Materials,* 4**,** 035016.

Afroj, S., Tan, S., Abdelkader, A. M., Novoselov, K. S. & Karim, N. 2020. Highly Conductive, Scalable, and Machine Washable Graphene-Based E-Textiles for Multifunctional Wearable Electronic Applications. *Advanced Functional Materials*.

Bae, J., Song, M. K., Park, Y. J., Kim, J. M., Liu, M. & Wang, Z. L. 2011. Fiber supercapacitors made of nanowire-fiber hybrid structures for wearable/flexible energy storage. *Angewandte Chemie - International Edition*.

Chen, Q., Li, X., Zang, X., Cao, Y., He, Y., Li, P., Wang, K., Wei, J., Wu, D. & Zhu, H. 2014. Effect of different gel electrolytes on graphene-based solid-state supercapacitors. *RSC Advances,* 4**,** 36253-36256.

Chen, T. & Dai, L. 2016. Flexible and wearable wire-shaped microsupercapacitors based on highly aligned titania and carbon nanotubes. *Energy Storage Materials,* 2**,** 21-26.

Li, X., Liu, R., Xu, C., Bai, Y., Zhou, X., Wang, Y. & Yuan, G. 2018. High-Performance Polypyrrole/Graphene/SnCl2 Modified Polyester Textile Electrodes and Yarn Electrodes for Wearable Energy Storage. *Advanced Functional Materials,* 28**,** 1800064.

Liu, C., Zhao, S., Lu, Y., Chang, Y., Xu, D., Wang, Q., Dai, Z., Bao, J. & Han, M. 2017. 3D Porous Nanoarchitectures Derived from SnS/S-Doped Graphene Hybrid Nanosheets for Flexible All-Solid-State Supercapacitors. *Small,* 13**,** 1603494.

Liu, L., Tian, Q., Yao, W., Li, M., Li, Y. & Wu, W. 2018. All-printed ultraflexible and stretchable asymmetric in-plane solid-state supercapacitors (ASCs) for wearable electronics. *Journal of Power Sources,* 397**,** 59-67.

Liu, S., Xie, J., Li, H., Wang, Y., Yang, H. Y., Zhu, T., Zhang, S., Cao, G. & Zhao, X. 2014. Nitrogen-doped reduced graphene oxide for high-performance flexible all-solid-state micro-supercapacitors. *Journal of Materials Chemistry A,* 2**,** 18125-18131.
